# The Spread of the Covid-19 Pandemic in Time and Space

**DOI:** 10.3390/ijerph17113827

**Published:** 2020-05-28

**Authors:** Christian M. Hafner

**Affiliations:** Louvain Institute of Data Analysis and Modelling in Economics and Statistics, Institute of Statistics, Biostatistics and Actuarial Sciences, Université Catholique de Louvain, Voie du Roman Pays, 20, 1348 Louvain-la-Neuve, Belgium; christian.hafner@uclouvain.be

**Keywords:** corona virus, spatial autoregressions, stochastic cycles, contagion, LASSO, networks

## Abstract

As the COVID-19 pandemic has had a profound impact on public health and global economies in 2020; it is crucial to understand how it developed and spread in time and space. This paper contributes to the growing literature by considering the dynamics of country-wise growth rates of infection numbers. Low-order serial correlation of growth rates is predominantly negative with cycles of two to four days for most countries. The results of fitted spatial autoregressive models suggest that there is high degree of spillover between countries. Forecast variances of many countries, in particular those with a high absolute number of infections, can to a large extent be explained by structural innovations of other countries. A better understanding of the serial and spatial dynamics of the spread of the pandemic may contribute to an improved containment and risk management.

## 1. Introduction

On December 31, 2019, the first case of a pneumonia caused by a new type of coronavirus was reported to the World Health Organization (WHO) office in Wuhan, China. Since then, the new disease, named COVID-19 on February 11, 2020, by the WHO, has developed and spread quickly throughout the world, touching essentially all countries by the end of April 2020, despite numerous containment strategies, social distancing, and the closing of borders.

The lockdown in many countries led to economic recessions associated with a slump of economic activity, rise of unemployment, and high uncertainty on financial markets. As shown, e.g., by Li et al. (2020), Wang et al. (2020), and Zhang and Ma (2020) [1,2,3], the pandemic had enormous psychological consequences for the population. Risk management has become essential during times of crises, and McAleer (2020) emphasizes the importance of prevention relative to the cure [4]. The outbreak and evolution of the COVID-19 pandemic has been analyzed from different perspectives. For example, Li and Linton (2020) fit country-wise quadratic regressions to estimate the peak periods [5]. Yue et al. (2020) analyze the impact of the pandemic on China’s economy [6], and Wang et al. (2020) consider the risk management of COVID-19 by universities in China [7]. Yue et al. (2020) propose solutions and recommendations related to early warning, identification, and monitoring of risks [8]. Liu et al. (2020) analyze the Chinese experience and its implications for other countries [9]. Chang et al. (2020) present a charter for a sustainable travel, tourism, and hospitality industry for the time after COVID-19 [10]. Chang and McAleer (2020) critically evaluate the Global Health Security Index (2019) [11], which provides data before the discovery of COVID-19 and makes it possible to evaluate how countries might have been prepared for a pandemic and acted accordingly. Further research analyzing the containment strategies of individual countries and global analysis of the COVID-19 situation includes Wang and Wang (2020), Zhao and Chen (2020), Han et al. (2020), and Di Gennaro et al. (2020) [12,13,14,15].

Despite the growing literature, there seems to be a gap in the understanding how the pandemic evolved over time, and how it spread to other countries. Rather than merely providing descriptive statistics, we would like to fit a joint model for the temporal and spatial dynamics that allows to measure the degrees of contagion between countries and yield predictions for the numbers of infected people. In this paper, we use spatial autoregressions to model daily growth rates of the number of incidences per country. Day-to-day relative changes in the number of incidences are informative about short-term effects of containment strategies and spillovers between countries. We refrain from modeling long-term trends in the data, which are notoriously difficult to capture, but rather focus on short-term dynamics, assuming stationarity for the investigated sample horizon.

It turns out that, perhaps surprisingly, there is a strong informational content in incidence growth rates, in the sense that they reveal negative autocorrelations and cyclical behavior. In a preliminary univariate analysis, we find strong negative low-order (i.e., mostly first order, but many even higher) autocorrelations of growth rates, as well as stochastic cycles of 2 to 4 days, meaning that fitted autoregressive models of order two have complex roots for the vast majority of countries. We embed these findings into a multivariate context, allowing for spillover (i.e., Granger causality in econometric terminology) between countries, as well as for spatial autocorrelation. The modeling framework is that of a spatial vector autoregression.

Spatial vector autoregressions have been used, e.g., by Wei (2015) to forecast influenza incidence rates of US states [16]. In a panel data context, similar models have been proposed by Elhorst (2003), Beenstock and Felsenstein (2007) and Lee and Yu (2012) [17,18,19]. Spatial vector autoregressions impose a structure on the covariance matrix of the error term that is motivated, e.g., by geographical factors such as distances. This structure enables a structural analysis of the vector autoregressive model (VAR) model that is not depending on a particular decomposition of the covariance matrix, a common problem in classical VAR models. In our case, we find that the best specification of the spatial weights matrix is based on inverse distances, modulated by population sizes.

We follow the approach of Diebold and Yilmaz (2014) to consider forecast error variance decompositions [20], interpreted as directed weighted networks. In our case, these decompositions depend on both the serial and spatial autocorrelation matrices. We find strong spillovers between countries in the sense that forecast error variances of many countries can, to a large extent, be explained by structural innovations of other countries. This seems to be particularly strong for countries with high absolute number of infections such as the USA, Italy, China, Germany, and Great Britain.

Our results indicate that geographical contagion of the COVID-19 pandemic has been and continues to be an important factor. The estimated spatial correlation is positive and highly significant, meaning that contagion is particularly strong between close and highly populated countries. These findings may have consequences for local and global containment and mitigation strategies.

## 2. Methodology and Results

We consider the daily number of newly infected people per country across time, as published by the European Centre for Disease Prevention and Control (ECSD), an agency of the European Union. Data are available for download at https://opendata.ecdc.europa.eu/covid19/casedistribution. In the early period of the epidemic, i.e., January and February of 2020, only few countries had significant numbers of infections, so we discard this period from the sample and consider the period of March and April, i.e., a sample size of 61 days. Population data are provided by the World Bank and are also available in the data sets provided by ECSD.

**Figure 1:**
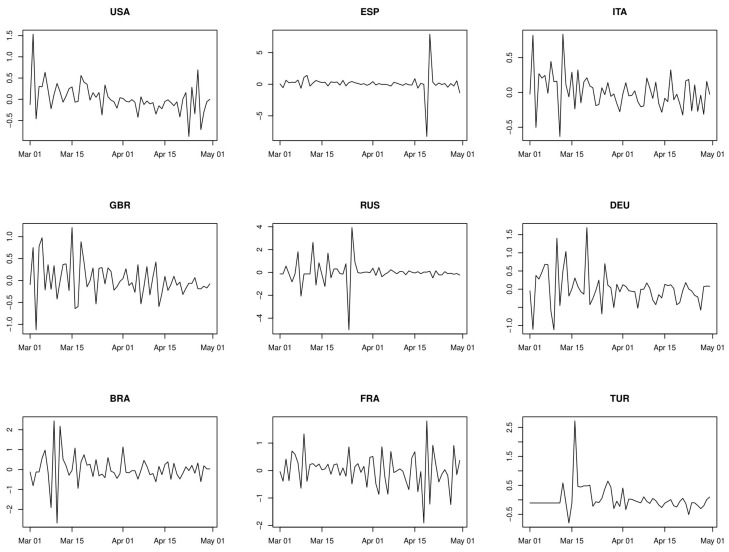
Growth rates of the nine countries with highest number of cases until end of April 2020: USA, Spain, Italy, Great Britain, Russia, Germany, Brazil, France, Turkey.

There are many alternatives to model the time trends and dynamic properties of the data, including quadratic trends as in Li and Linton (2020) [5]. We follow Yue et al. (2020) in considering autoregressions for the growth rates [6]. Let $Y_{it}$ be the number of newly infected people in country i,i=1,…,N, at time *t*, t=1,…,T. We model growth rates $y_{it}:=log\left(Y_{it}+1\right)-log\left(Y_{i,t-1}+1\right)$ via spatial autoregressive processes of order *p*, i.e.,

(1) $$y_{it}=\mu_{i}+\overset{p}{\underset{j=1}{\sum }}\alpha_{ij}\left(y_{i,t-j}-\mu_{i}\right)+\varepsilon_{it}$$

(2) $$\varepsilon_{it}=\rho \overset{N}{\underset{j=1}{\sum }}w_{ij}\varepsilon_{jt}+u_{it},\;u_{it}∼N\left(0,\sigma^{2}\right)$$

where $w_{ij}$ are spatial weights with $w_{ii}=0$, *i* = 1…, *N*, and $\mu_{i},\alpha_{ij}$ are coefficients. The weights could, for example, be inversely related to the distance $d_{ij}$ between countries *i* and *j*. Beenstock and Felsenstein (2007) suggest to modulate inverse distances with the relative population size of both countries [18], that is

(3) $$w_{ij}=\frac{1}{d_{ij}}\frac{Z_{j}}{Z_{i}+Z_{j}}$$

where $Z_{i}$ is the population of country *i*. Hence, bigger countries receive more weight than smaller countries. The growth rates of the nine countries with highest number of cases until end of April, 2020, are depicted in Figure 1.

The error term can be written in vector form, stacking it into an (*N* × 1) vector $\varepsilon_{t}$, as

(4) $$\varepsilon_{t}=\rho W\varepsilon_{t}+u_{t}$$

where *W* is the spatial weight matrix *W* with $w_{ii}=0$, *i* = 1…, *N*. An advantage of the spatial error model is that, once ρ is estimated, the structural shocks $u_{t}$ can readily be obtained by the transformation $\left(I_{N}-\rho W\right)\varepsilon_{t}$. This does not require decomposing a variance–covariance matrix as in classical VAR models, for which different methods are often a matter of debate in the empirical literature, see e.g., the discussion in Lütkepohl (2005) [21].

The dynamics of $y_{t}$ can be generalized to a vector autoregressive model (VAR) of the form

(5) $$y_{t}=\mu +\overset{p}{\underset{j=1}{\sum }}A_{j}\left(y_{t-j}-\mu \right)+\varepsilon_{t}$$

where $\varepsilon_{t}$ is a spatial error term as in Equation (4), and $A_{j}$ are *N* × *N* parameter matrices. For large *N*, restrictions are necessary for $A_{j}$ as otherwise there would be too many parameters to estimate for reasonable sample sizes. Indeed, in our case, the number of countries is larger than the number of days in the sample, so that methods are required that penalize model complexity.

To gain insight into the dynamics of infection growth rates, we will first look at univariate autoregressive models because it reveals some interesting dynamic features of the data. We will then consider spatial vector autoregressions to model the spatial and temporal relationships between countries. Of the more than 200 countries that have reported Covid-19 infections, we have selected the 100 countries with the highest number of infections as of end of April 2020. Thus, our cross-section dimension is given by *N* = 100. The reason to exclude countries with smaller number of cases is the highly erratic behavior of corresponding growth rates that could potentially bias the estimation of spatial correlation patterns.

### 2.1. Univariate Analysis

We fit autoregressive models of order p to the growth rate of infections for each country, where the lag order p is selected by the Akaike information criterion (AIC). The maximum lag chosen is 10, but for no country the selected p is larger than 2. Results are reported in Table A1 in the Appendix.

The empirical results suggest that:

1. First order serial correlations of growth rates are negative.
2. For the countries with AR(2) dynamics, there are stochastic cycles in growth rates with an average length of about three days.
3. The spatial autocorrelation coefficient ρ is strongly significant.

It is quite remarkable that except for one country (Andorra), all first order autocorrelation coefficients are negative with an average of −0.46. The second order autocorrelation coefficients are closer to zero, but still quite significant for many.

It turns out that among the 100 countries with the highest number of reported cases, 72 have growth rates with AR(2) dynamics, and of these all but one (Denmark) have roots of the characteristic equations that are complex, indicating the presence of stochastic cycles. The average length of these cycles is calculated as

(6) $$k_{i}=\frac{2\pi }{arccos\left(\alpha_{i1}/\left(2\sqrt{-\alpha_{i2}}\right)\right)}$$

All average cycle durations happen to be between 2 and 4 days, with an average of 2.8 days across countries. We do not have an explanation for the presence of such cycles, and whether they are genuine to the flux of new infections within a country, or artificially generated by the reporting practice. However, given that almost all autocorrelations of order larger than two tend to be small and often negligible, we can exclude seasonal or weekend effects as potential explanation. We next move on to the multivariate analysis in order to understand potential spillovers between countries.

### 2.2. Multivariate Analysis

**Figure 2:**
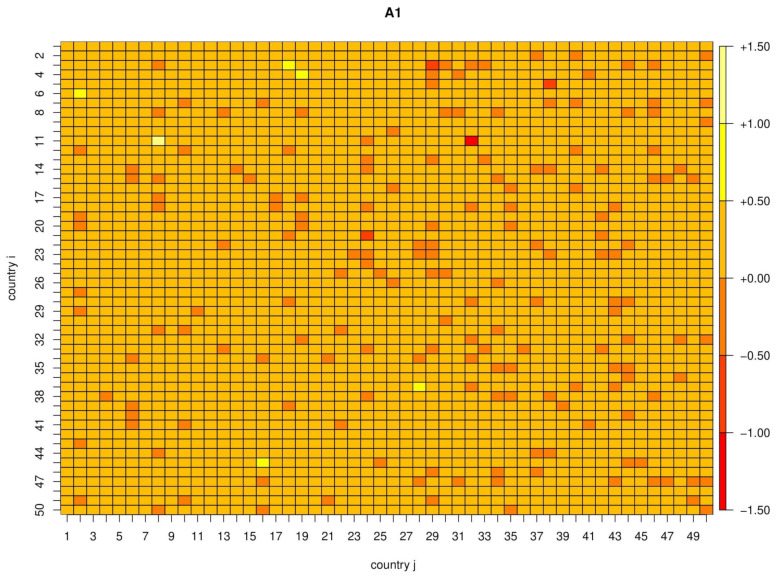
Estimated parameter matrix *A***_1_**.

**Figure 3:**
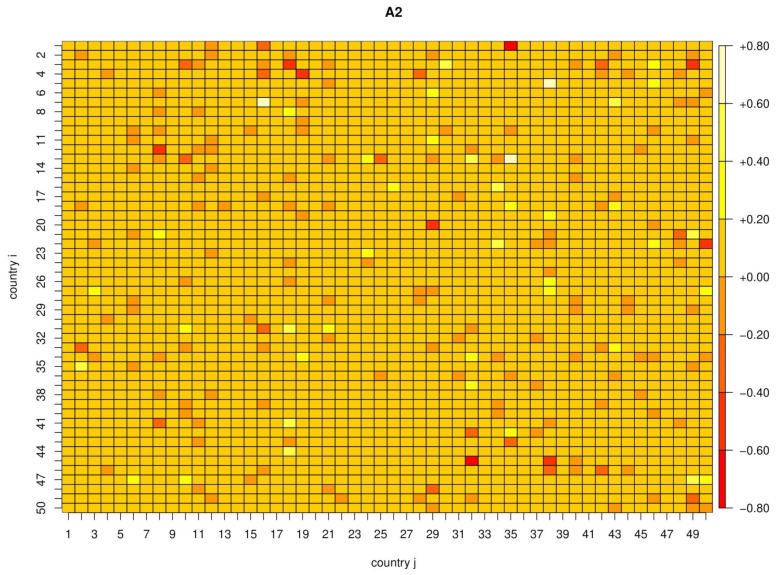
Estimated parameter matrix *A***_2_**.

For the specification of the VAR model in Equation (5), we first choose the lag order of *p* = 2, which corresponds to the maximum of the selected lag orders found for the univariate models. We then estimate the model by minimizing equation by equation the following criterion

(7) $$\overset{T}{\underset{t=1}{\sum }}\varepsilon_{it}^{2}+\lambda_{i}\overset{N}{\underset{j=1}{\sum }}\overset{2}{\underset{k=1}{\sum }}\left|A_{ijk}\right|$$

with respect to the coefficients $A_{ijk}$, where $\lambda_{i}$ is chosen by ten-fold cross-validation. This criterion is known in statistics as the least absolute shrinkage and selection operator (LASSO), see e.g., Tibshirani (1996) [22]. Figure 2 and Figure 3 show the parameter estimates for the 50 × 50 subset of countries with most infections. The number of non-zero coefficients in $A_{1}$ by country ranges from 1 to 32 (out of 100), while the total number is 1741 (out of 10,000). Similarly, the number of non-zero coefficients in $A_{2}$ ranges from 6 to 35, with a total number of 1614 non-zero coefficients.

In a second step, the coefficient ρ of the spatial error component model in Equation (4) is estimated by Gaussian maximum likelihood, see e.g., Bivand et al. (2013) [23]. The model obviously depends on the specification of the spatial weights matrix *W*, for which we used inverse distances modulated by the relative population sizes, as in Equation (3). Distances are measured as the geographic distance in kilometers on the WGS ellipsoid between the centroids of two countries. This specification led to the highest likelihood among a set of alternative specifications including unweighted inverse distances, *k*-nearest neighbor and contiguity matrices. We obtain an estimate of 0.2805 with associated standard error of 0.0518, which suggests that spatial correlation is highly significant and important to be included in the subsequent analysis. After estimation, the residuals $u_{t}$ have been tested for remaining spatial autocorrelation using the Baltagi et al. (2007) spatial autocorrelation test for panel models [24], which is an extension of the classical Moran test. The *p*-value of the test is 0.157, which suggests that spatial autocorrelation has been sufficiently captured by the model.

In the following, we study the implications of the estimated parameters for the decompositions of the variances of forecast errors, in the spirit of Diebold and Yilmaz (2014) [20]. This allows quantifying the network relationships between countries. Starting from the reduced form VAR (2) model in Equation (5) with spatial error terms $\varepsilon_{t}$ in Equation (4), we may obtain the infinite order vector moving average (VMA) representation,

(8) $$y_{t}=\overset{\infty }{\underset{j=0}{\sum }}\Phi_{j}\varepsilon_{t-j}$$

with coefficient matrices $\Phi_{j}$ that, for our VAR (2) model, can be obtained recursively as $\Phi_{0}=I_{N}$, $\Phi_{1}=A_{1}$, $\Phi_{2}=\Phi_{1}A_{1}+A_{2},\ldots ,\Phi_{j}=\Phi_{j-1}A_{1}+\Phi_{j-2}A_{2},\ldots$, see e.g., Lütkepohl (2005) [21].

From the spatial error model in Equation (4) we then obtain orthogonalized shocks as $u_{t}=\left(I_{N}-\rho W\right)\varepsilon_{t}$ and rewrite the model as

(9) $$y_{t}=\overset{\infty }{\underset{j=0}{\sum }}\Theta_{j}u_{t-j}$$

where $\Theta_{j}:=\Phi_{j}{\left(I_{N}-\rho W\right)}^{-1}$. Thus, having orthogonalized the error terms, we can perform structural analysis, and this is independent of a particular decomposition of a variance-covariance matrix, which is a common problem in classical VAR models. The proportion of the *h*-step forecast error variance of country *i* accounted for by innovations in country *j* is then given by

(10) $$\omega_{ij,h}=\overset{h-1}{\underset{k=0}{\sum }}\theta_{ij,k}^{2}/\overset{N}{\underset{j=1}{\sum }}\overset{h-1}{\underset{k=0}{\sum }}\theta_{ij,k}^{2}$$

where $\theta_{ij,k}$ is the *ij*-th element of the parameter matrix $\Theta_{k}$. It is important to emphasize that $\omega_{ij,h}$ depends on both the dynamics of the VAR system via the parameter matrices $\Phi_{j}$, and the spatial lag matrix, $\left(I_{N}-\rho W\right)$. Hence, forecast variance decompositions are determined by spatial and temporal dynamics jointly, which corroborates the importance of a joint spatio-temporal modeling approach.

**Figure 4:**
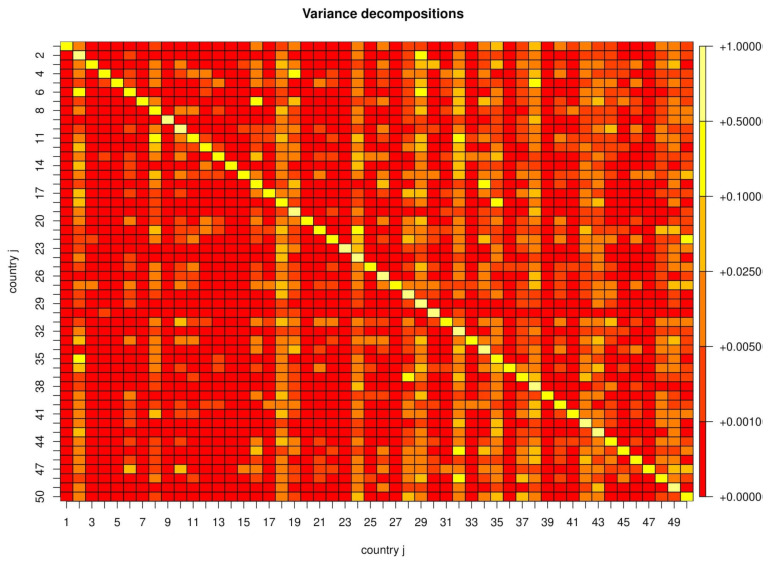
Decompositions of forecast variances.

The proportions $\omega_{ij,h}$ are reported graphically in Figure 4 for the 50 countries having the highest number of infections. We chose a forecast horizon of *h* = 10, which for our parameter estimates is almost identical to long-run forecasts (h=∞) as $\omega_{ij,h}$ converges quickly to a constant as *h* increases. Not surprisingly, most countries’ forecast errors are explained to a good extent by their own history, i.e., the diagonal elements of ω tend to be larger than the off-diagonal elements. The average of the diagonal elements is 0.47 with a maximum of 0.96 and a minimum of 0.1. This also implies that there are many countries for which the sum of the contributions of other countries is larger than that of their own history.

**Figure 5:**
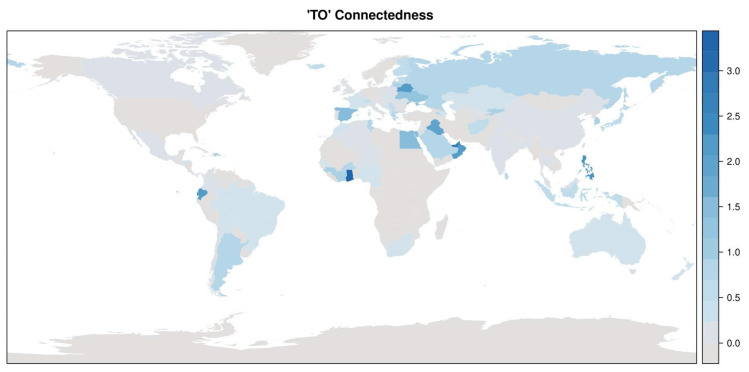
Directional “TO” connectedness measure, or “TO”-degrees.

**Figure 6:**
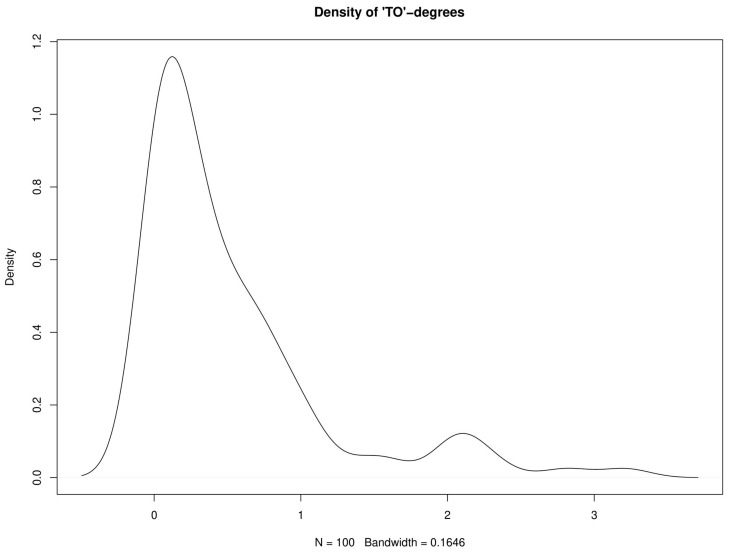
Kernel estimate of the density of “TO”-degrees.

This analysis can be refined following Diebold and Yilmaz (2014) by viewing variance decompositions as weighted directed networks [20]. They define two directional connectedness measures, “to” and “from”, for each country. The “to” measure is defined as

(11) $$\omega_{\cdot j}:=\overset{N}{\underset{i=1,i\neq j}{\sum }}\omega_{ij}$$

where we suppress the *h*-index for simplicity. The $\omega_{\cdot j}$ measures the sum of the contributions of country *j* to all other countries’ forecast errors, and it can be viewed as a “to”-degree of a node (i.e., a country) of the network. The support of the univariate distribution of $\omega_{\cdot j}$ is $\left[0,N\right]$. The countries’ “to”-degrees are visualized in Figure 5, and the corresponding distribution in Figure 6. The distribution of “to”-degrees has a bi-modal structure, where the majority of “to”-degrees is close to zero, and a small group of countries having “to”-degrees of 2 or larger. The countries with highest “to”-degrees are Ghana, United Arab Emirates, Philippines, Ecuador, Oman, Belarus, Singapore, Iraq, Spain, and Egypt. Note that each continent has at least one country with a high “to”-degree, i.e., a country whose innovations in infection rates help to predict infection rates of other countries.

Likewise, the “from” directional connectedness is defined as

(12) $$\omega_{i\cdot }:=\overset{N}{\underset{j=1,j\neq i}{\sum }}\omega_{ij}$$

**Figure 7:**
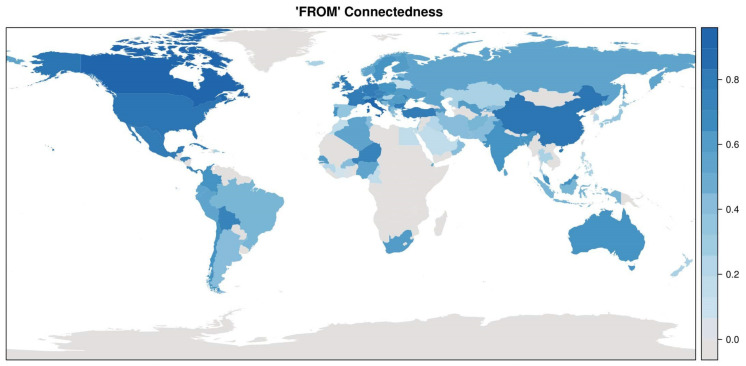
Directional “FROM” connectedness measure, or “FROM”-degrees.

**Figure 8:**
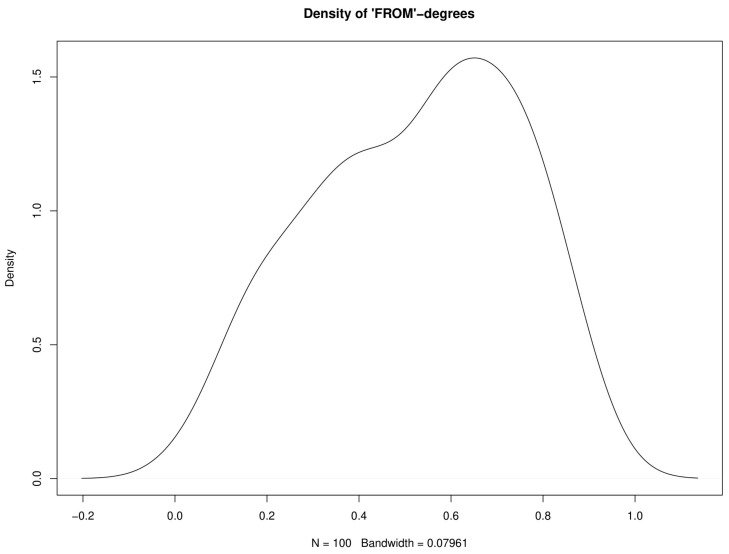
Kernel estimate of the density of “FROM”-degrees.

And measures the sum of the contributions of all other countries *j* to explain the forecast error variance of country *i*. The support of the univariate distribution of $\omega_{i\cdot }$ is $\left[0,1\right]$. These measures are visualized in Figure 7, and the corresponding distribution in Figure 8. Note that, by construction, the means of the “to” and “from” distributions are the same, 0.5222, but that the “from” distribution is more concentrated around values close to one, meaning that there are many countries whose forecast variances can to a large proportion be explained by the innovations of other countries. The countries with highest “from”-degrees are Canada, Italy, USA, China, Belgium, Bulgaria, Mexico, Germany, Great Britain, and Denmark. Note that these countries tend to have high absolute number of infection cases. Note also that the proportion of developing and emerging countries among those with high “to”-degrees is larger than among those with high “from”-degrees.

Finally, the total connectedness measure for the network of countries is given by $\omega :=\sum_{i=1}^{N}\omega_{i\cdot }=\sum_{j=1}^{N}\omega_{\cdot j}$, which corresponds to the mean degree of the network. We obtain a mean degree of 52.22.

## 3. Conclusions

Our findings suggest that daily growth rates of new infections per country are negatively autocorrelated and show stochastic cycles of about two to four days. In a spatial vector autoregression, we find high spillovers between countries, motivated by highly significant serial and spatial autocorrelations. Forecast error variances are to a large extent explained by other countries’ innovations. This holds especially for countries with high absolute numbers of infections.

High contagion degrees between countries requires risk and crisis management that takes the evolution of the pandemic in other countries into account. We have seen the pandemic starting to develop in China, then spreading to Europe and the USA, and finally, developing strongly in countries such as Russia and Brazil. This paper contributes to a better understanding of how the crisis has evolved both over time and across countries, which may be fruitful for an improved risk management of future crises.

## Appendix

**Table A1:** Estimation results for the univariate AR(*p*) models.

	Country	Mu	Alpha1	Alpha2	K	Acf1	Acf2
1	USA	0.1190	NA	NA	NA	−0.5812	0.1025
2	ESP	0.0551	−0.6357	−0.3070	2.879772	−0.4078	−0.0964
3	ITA	0.0197	−0.2187	NA	NA	−0.3080	−0.1204
4	GBR	0.0994	−0.2747	−0.3170	3.457548	−0.4656	−0.1669
5	DEU	−0.0483	NA	NA	NA	−0.7526	0.4425
6	FRA	0.0591	−0.5001	NA	NA	−0.5180	0.0404
7	TUR	0.1290	0.2093	NA	NA	−0.4603	0.1770
8	RUS	0.1454	−0.6229	−0.3060	2.897091	−0.2381	−0.1603
9	IRN	0.0153	−0.7193	−0.3499	2.824746	−0.3826	−0.2127
10	BRA	0.1456	−0.5581	NA	NA	−0.7432	0.4734
11	CAN	0.0951	−0.3906	NA	NA	−0.4648	−0.0175
12	BEL	0.0951	NA	NA	NA	−0.3717	−0.1870
13	NLD	0.0705	NA	NA	NA	−0.1379	0.0532
14	PER	0.1315	−0.7798	−0.3740	2.777552	−0.4865	−0.0313
15	IND	0.1246	−0.7751	−0.1902	2.357376	−0.4758	−0.1840
16	CHE	0.0532	−0.6605	−0.1818	2.557440	−0.5581	0.1938
17	PRT	0.1035	−0.4378	NA	NA	−0.2475	−0.1414
18	ECU	0.0618	−0.3215	−0.3590	3.410295	−0.6846	0.3053
19	SAU	0.1182	−0.7888	−0.5028	2.908032	−0.5923	0.0215
20	SWE	0.0980	−0.2257	NA	NA	−0.3906	0.0849
21	IRL	0.0965	−0.3648	NA	NA	−0.4258	0.0670
22	MEX	0.1077	−0.3284	−0.1960	3.220894	−0.4257	−0.0989
23	PAK	0.1142	−0.6143	−0.3125	2.919015	−0.5396	0.1344
24	SGP	0.0764	−0.6688	−0.3331	2.870741	−0.4410	0.0195
25	CHL	0.1154	−0.4258	NA	NA	−0.5679	0.1749
26	ISR	0.0548	−0.8392	−0.2685	2.498557	−0.4177	0.1557
27	AUT	0.0481	−0.2929	−0.2300	3.340180	−0.5234	0.3204
28	JPN	0.0409	−0.3556	−0.3084	3.312745	−0.6871	0.3177
29	BLR	0.1105	−0.5120	−0.3774	3.140573	−0.2509	−0.0071
30	QAT	0.0925	−0.8442	−0.3308	2.623628	−0.2274	0.2722
31	POL	0.0897	−0.4912	NA	NA	−0.7159	0.4200
32	ARE	0.1035	−0.9655	−0.4536	2.651255	−0.4971	0.0442
33	ROU	0.0913	−0.4175	NA	NA	−0.2366	−0.2829
34	IDN	0.0779	−0.7072	−0.3386	2.825275	−0.4945	−0.1337
35	UKR	0.0000	−0.5586	−0.3737	3.071974	−0.3739	0.0234
36	DNK	0.0709	−0.1745	0.2325	NaN	−0.5398	−0.0478
37	SRB	0.0927	−0.5346	−0.3479	3.078214	−0.5001	0.1388
38	PHL	0.0922	−0.8493	−0.3343	2.622693	−0.0336	−0.0254
39	NOR	0.0357	−0.7009	−0.4292	2.942658	−0.6548	0.2291
40	CZE	0.0534	−0.2509	NA	NA	−0.5757	0.3656
41	BGD	0.1039	−0.6032	−0.2978	2.913854	−0.6575	0.1833
42	KOR	−0.0693	−0.7663	−0.3236	2.720150	−0.5332	0.0641
43	DOM	0.0833	−0.6311	−0.2695	2.825041	−0.5545	0.0766
44	AUS	0.0237	−0.4603	NA	NA	−0.4901	−0.0438
45	PAN	0.0827	−0.3611	NA	NA	−0.6513	0.3146
46	COL	0.0933	−0.6590	−0.2212	2.677165	−0.5283	0.0350
47	MYS	0.0402	−0.8145	−0.4581	2.834799	−0.5329	0.0334
48	ZAF	0.0934	−0.7034	−0.2244	2.609739	−0.6558	0.2550
49	EGY	0.0804	−0.7420	−0.5007	2.959918	−0.3648	0.2310
50	FIN	0.0510	−0.7983	−0.4787	2.874698	−0.6616	0.2868

**Notes:** Countries are indexed by their ISO3 code. The number mu is the average growth rate. AR orders were selected by the AIC criterion. If *p* = 0, then both *α*_1_ and *α*_2_ are marked as NA, if *p* = 1, then *α*_2_ is marked as NA. The number *k* gives the average length of the stochastic cycles, in days, for those AR(2) models that have complex roots. For all other models, *k* is marked as NA; acf1 and acf2 are autocorrelation coefficients of order 1 and 2 for the growth rates.
